# Healthcare Provider Narratives of the Impacts of the COVID-19 Pandemic on Pregnant and Parenting Youth in Canada: A Qualitative Study

**DOI:** 10.3390/ijerph21111419

**Published:** 2024-10-26

**Authors:** Salima Meherali, Mariam Ahmad, Amyna Ismail Rehmani, Amber Hussain, Saba Nisa, Simone Lebeuf, Sarah Munro, Chandra Ashton, Zohra S. Lassi, Ashley Vandermorris, Hasina Samji, Wendy V. Norman

**Affiliations:** 1 Faculty of Nursing, College of Health Sciences, University of Alberta, Edmonton, AB T6G 1C9, Canada; mariam2@ualberta.ca (M.A.); amynaism@ualberta.ca (A.I.R.); ahussai4@ualberta.ca (A.H.); snisa@ualberta.ca (S.N.); 2 Department of Pediatrics, University of Alberta, Edmonton, AB T6G 1C9, Canada; simone.lebeuf@albertahealthservices.ca; 3 Department of Health Systems and Population Health, School of Public Health, University of Washington, Seattle, WA 98195, USA; sarahmun@uw.edu; 4 Terra Centre, Edmonton, AB T5N 3A2, Canada; cashton@terracentre.ca; 5 Faculty of Health and Medical Sciences, School of Public Health, University of Adelaide, Adelaide, SA 5005, Australia; zohra.lassi@adelaide.edu.au; 6 Robinson Research Institute, University of Adelaide, Adelaide, SA 5005, Australia; 7 Department of Paediatrics, University of Toronto, 555 University Ave, Toronto, ON M5G 1X8, Canada; ashley.vandermorris@sickkids.ca; 8 Faculty of Health Sciences, Simon Fraser University, Burnaby, BC V5A 1S6, Canada; hsamji@sfu.ca; 9 Department of Family Practice, Faculty of Medicine, University of British Columbia, Vancouver, BC V6T 1Z4, Canada; wendy.norman@ubc.ca

**Keywords:** COVID-19 pandemic, pregnant youth, parenting youth, healthcare providers, service provision

## Abstract

The COVID-19 pandemic led to significant challenges for healthcare providers working with pregnant and parenting youth. However, the impacts of the pandemic on this population and healthcare services from the perspective of healthcare providers are not well documented in Canada. We examined the narratives and experiences of healthcare providers regarding these impacts and explored the challenges to service provision. Using a qualitative interpretative description (ID) approach, we recruited 25 health and service providers from Alberta, Ontario, and British Columbia for individual qualitative interviews. Our analysis resulted in three themes: complexities of health service provision during COVID-19, healthcare providers’ accounts of impacts on pregnant and parenting youth, and leveraging challenges into opportunities for service provision. Participants described the influence of pandemic policies and distancing measures on accessibility of health services, availability of healthcare resources and personnel, and well-being of their clients. They also reported increased mental health issues, isolation, and exacerbation of inequities within this population. Providers highlighted the role of telemedicine in ensuring some degree of continuity of care. Additionally, they commented on service adaptations to address the evolving needs of their clients. Our findings underline the need for a resilient and adaptable healthcare system that can better support the needs of vulnerable populations during crises.

## 1. Introduction

The COVID-19 pandemic profoundly impacted health care and community service providers and necessitated rapid adaptations to ensure the continuity of essential services [1,2]. In Canada, healthcare workers faced challenges, such as increased workloads, heightened stress levels, and significant disruptions in routine care services, such as maternal and child health services [3,4]. Studies reporting on the active stages of the pandemic highlighted significant disruptions in perinatal care provision, with changes in service delivery, reductions in face-to-face appointments, and alterations in practice guidelines [5,6,7]. Service and healthcare providers, particularly those supporting vulnerable populations, experienced significant challenges during the initial stages, including rapid transition to virtual care, redeployments, and higher demand for services [1,8,9,10]. Many healthcare facilities were overwhelmed or repurposed for COVID-19 treatment, reducing routine perinatal care availability. This disruption might have resulted in missed or delayed diagnoses of complications such as gestational diabetes, preeclampsia, fetal growth restrictions, and other conditions that required timely intervention [11,12].

There is now increasing availability of evidence on the impacts that the pandemic and its associated policies had on perinatal care provision and maternal health outcomes. Systematic reviews by Townsend et al. [13] and Chmielewska et al. [14] reported global implications of the pandemic on maternal health outcomes with significant increases in maternal and fetal mortality. Among the populations most impacted by these outcomes were pregnant and parenting youth, as factors such as cost of services, remote living, low maternal age, and having a minority or an immigrant status were found to have a significant impact on the ability to navigate perinatal services during the pandemic [15]. Pregnant and parenting youth already facing unique challenges and with an increased risk of obstetric complications, experienced additional vulnerabilities concerning financial stability, social relationships, and accessing quality care [9,10]. The pandemic led to increased physical and mental health issues among this group, with higher incidents of anxiety, depression, loneliness, and broader socio-economic pressure [16,17].

Although a number of studies have documented the pandemic’s impacts on healthcare systems, workers, and maternal care provision (e.g., higher burnout, physical, emotional, and mental health issues) [1,2,8], there is limited evidence with regards to service provision to pregnant and parenting youth in Canada, particularly from the point of view of care providers. Therefore, we aimed to explore the challenges faced by healthcare and service providers offering services to pregnant and parenting youth, and their narratives of the impacts of pandemic response measures on their client populations. Specifically, our objectives were:

1. To understand the wider health impacts of the COVID-19 pandemic on pregnant and parenting youth from the providers’ perspective.
2. To understand the impact of public health measures on access to health services and social support services by pregnant and parenting youth during the COVID-19 pandemic.
3. To identify and recommend the formulation of healthcare policy and practice interventions to ameliorate the wider impacts of the COVID-19 pandemic on the health and well-being of pregnant and parenting youth

The perspectives of healthcare and service providers offer valuable insights into the broader consequences of COVID-19 on pregnant and parenting youth, shedding light on the complexities of navigating pregnancy, parenthood, and healthcare amidst a global health crisis. From these explorations we intend to propose effective interventions (policy, practice, and/or models of care) to mitigate the challenges faced by pregnant and parenting youth in order to be better prepared for future health emergencies and pandemics. This includes, but is not limited to, utilizing the knowledge generated through these narratives to develop a future pandemic response framework (national advocacy toolkit) that would aid in supporting pregnant and parenting youth and their children, and address the issues that healthcare and service providers advocate for their care groups.

## 2. Methods

### 2.1. Study Design

We conducted a national qualitative study across Canada using an interpretive description approach. An interpretive description (ID) methodology was appropriate for our study due to its exploratory nature, intending to facilitate the generation of insights pertinent to clinical practice [18,19]. Moreover, ID provides a flexible and accessible approach to analyzing data and generating knowledge from participants’ diverse subjective experiences and perceptions [19]. Our objective was to explore the impacts of the COVID-19 pandemic on pregnant and parenting youth from the viewpoint of service providers, aiming to acquire knowledge to guide the development of strategies addressing the distinct needs of this vulnerable demographic. We conducted individual qualitative interviews with service providers (i.e., nurses, midwife, physicians, pharmacist, social worker, psychologist, nutritionist) to explore the challenges they faced in providing services and the wider and long-term impacts of the pandemic on their client demographic. We asked open-ended questions, focusing on the influence of the pandemic on the provision of care to this group, the physical, mental, and psychological health impacts reported among their clients, and the challenges faced while providing services during the pandemic (see Supplementary File).

### 2.2. Participants and Recruitment

We used purposeful sampling to recruit participants who provided care or services to pregnant or parenting youth (aged 15–24 years) and their children from Alberta, Ontario, and British Columbia. We ensured that service providers from various disciplines and specialties were included to obtain a holistic understanding of the impacts on different aspects of pregnant and parenting youth’s lives. Purposeful sampling helped us deliberately choose participants who would provide rich data and comprehensive information based on their experience working with pregnant and parenting youth [19]. We used various strategies to reach out to potential participants, particularly through email listservs of partner organizations, social media advertisements, and snowball sampling.

### 2.3. Data Collection

We started data collection in September 2023, which concluded in February 2024. We used a semi-structured interview guide, which included open-ended questions regarding service provider’s experiences and perceptions of the impact of the pandemic on the well-being of pregnant and parenting youth and the challenges to service provision. We developed and adjusted the interview guide based on the responses and findings from the interviews in order to comprehend the identified patterns thoroughly. Data collection continued until we achieved saturation and informational redundancy, signifying the point at which participants no longer provided new information for the comprehensive description of the phenomenon [18,20].

Research assistants (Amber Hussain, Saba Nisa) and project coordinator (Mariam Ahmad) mentored by the PI (Salima Meherali), with extensive expertise in qualitative methodology, obtained informed consent from all participants, conducted the interviews, and collected sociodemographic information through Google Forms. The interviews were conducted online via Zoom and lasted approximately 30–60 min. All the interviews were audio-recorded, de-identified, and transcribed verbatim. Throughout the interviews, we kept a reflective log of the overall interview experience, key findings and discussion topics, and participants’ responses during the interviews.

### 2.4. Data Analysis

Following ID and thematic analysis principles, we analyzed the data concurrently with data collection and used a structured process to interpret findings and thematically analyze the data [19]. First, the team (A.H., M.A., S.N.) individually read the interview transcripts to familiarize themselves with the data, which allowed us to gain an initial understanding of recurring ideas and concepts. We identified patterns and keywords directly derived from the data. We used the qualitative analysis software NVivo version 14 to assist in organizing, coding, and managing the data. Next, we used NVivo to systematically code the data segments pertinent to the research objectives. Each member individually coded sections of the transcripts in NVivo, which helped us to obtain an unbiased opinion of how the data should be coded and categorized. By comparing codes and discussing preliminary categories, we identified discrepancies and refined our coding framework. Then, we organized our codes into preliminary themes, which we discussed with the whole team. We finalized the themes after a thoughtful discussion of the findings, preliminary themes, and reflective logs. 

### 2.5. Rigour

We used the trustworthiness criteria outlined by Lincoln and Guba [21]. We employed multiple triangulation methods to ensure the rigour of our findings. In addition to member checking, we used reflective logs to capture researchers’ observations and reflections throughout the interviews. Team discussions were held during data analysis to compare coding and interpretations, allowing for the refining of emerging themes. These diverse approaches helped validate the findings and enhance the credibility of our results. To ensure transferability, we provided detailed descriptions of the research context and participants’ attributes, enhancing the comprehension and applicability of the findings across various settings. For reliability, we conducted an iterative process of continuous code comparison and, to ensure confirmability, we conducted an audit trail and used a logbook.

## 3. Results

### 3.1. Participant Characteristics

We recruited a total of 25 participants from three provinces across Canada. The demographic characteristics of the participants are presented in Table 1.

### 3.2. Key Findings

We categorized our findings into three broad themes; the descriptions of each theme with their respective sub-themes are presented below.

#### 3.2.1. Complexities of Health Service Provision During COVID-19

We explored the challenges healthcare providers encountered while caring for pregnant and parenting youth during the pandemic, posed by redeployment, limited supplies, and the temporary closure of their workplace settings, and how it impacted their ability to reach out and provide care to youth. The scarcity of healthcare workers and resources impacted the delivery of healthcare services, particularly in maintaining the continuity of care for pregnant youth.

Navigating increased workload with additional measures

Healthcare providers shared their experiences with unexpected changes in placements and redeployment of staff to essential COVID-19 operations. Many participants reported that they were re-deployed to work in COVID-19 units, call centres, and immunization clinics, leaving behind fewer professionals to cater to the regular services for pregnant and parenting youth. This redeployment and diversion of resources increased the workload for the healthcare professionals who were working in their original settings. One participant highlighted how redeployment impacted their team and the availability of services for youth:

We’re a very small team. There’s only five nurses on our team, and during the pandemic, we were redeployed to immunize… two nurses were off, so there were actually only three of us… we just left these moms hanging, hoping that they would connect to their doctors.

(P-08, nurse)

Participants felt overwhelmed and burned out due to the increased workload, while maintaining COVID-19 precautions and additional measures to prevent transmission: “I think we burnt out a lot of our nurses during the pandemic, just by doing redeployment. But the thing is, we didn’t have any choice, either” (P-14, physician). Healthcare providers reflected on their collective experiences of emotional exhaustion, fatigue, and mental health concerns:

Everyone else was at home in their safe little bubbles, but we were going to work, and we were still seeing the public and the guidelines of how to do that safely because of supply issues, and what we had available, it seemed to change so often that healthcare workers were always stressed out and fatigued.

(P-01, nurse)

The participant further commented on how additional protective measures introduced during the pandemic further increased the workload and consumed more time than usual:

I would say time. Again, it just takes us so much more time, because you have to have that conversation with the patient on the phone, but then you’d have to fax that prescription. You’d have to fax that bloodwork. So there’s just extra steps that we wouldn’t normally have, so in terms of workload, it was more.

(P-01, nurse)

Providing care with limited resources and support

Healthcare providers narrated their experiences of providing care with limited resources and the challenges of identifying sources of support for their patients. Many participants reported difficulties in consistently enforcing safety protocols, such as continuously wearing masks and encouraging clients to do the same. One participant emphasized the complexities of these measures while dealing with an influx of patients amidst an inadequate supply of PPE:

Like all the COVID measures, mask up, washing hands, making sure the patients are also doing that, observing these measures were work by itself. At times the equipment was not enough. The PPEs were not enough. It was difficult.

(P-04, pharmacist)

Several providers shared how they were unable to provide opportunities for adequate support to promote comfort and care for pregnant youth, particularly during childbirth:

The COVID restrictions only allowed one person to be in the room during delivery, so that was a barrier for people who wanted the support. Sometimes appointments, even ultrasounds, nobody was allowed to enter with them, so that also makes it hard, especially if this is their first time going through the process and they have to kind of do it alone.

(P-07, nurse)

Lack of trust in and comfort with health services

The impacts of the pandemic were not only felt in the delivery of services but also in how these services were perceived by pregnant and parenting youth. Participants noticed a drastic change in the level of trust that their clients had in the healthcare system, which was evident by the reduction in the number of individuals accessing their services:

Their [youth] trust in healthcare isn’t what it used to be. Our numbers went way down. We used to run groups in different areas of the city, up to 50, and now those groups are struggling to have like one to five people.

(P-09, nurse)

Participants highlighted that working with youth, especially those who are marginalized or vulnerable, requires special attention and time to effectively respond to their unique needs. During the pandemic, building connections and effective relationships with youth became more difficult with additional distancing measures and barriers:

Feeling a connection with youth was a lot more difficult. Working with young folks, especially those who have been disadvantaged, marginalized or gone through foster care, it takes time and showing up consistently to build that relationship. With the pandemic, resource limitations, not being able to meet face to face, these set up more barriers to build that initial connection with folks.

(P-10, social worker)

Moreover, with remote consultations, healthcare providers expressed the challenges of forming therapeutic relationships with clients: “It’s difficult to develop trusting relationships with care providers when you can’t see them in person.” (P-21, psychologist). Participants communicated their inability to meet clients and adequately support their needs, which impacted their care outcomes and heightened communication barriers:

Some clients, I never actually met in person. We would just talk on the phone, and I would mail them things, or drop them off on their doorstep, and it’s hard to trust someone if you don’t actually meet them. And then once they delivered their baby, they were home alone, and it’s very difficult to help support someone with breastfeeding and postnatal care over the phone.

(P-09, nurse)

#### 3.2.2. Healthcare Providers’ Accounts of Impacts on Pregnant and Parenting Youth

We inquired with healthcare providers about their perceptions of the impacts of the COVID-19 pandemic on pregnant and parenting youth. Participants highlighted the challenges that the youth encountered, especially those with existing vulnerabilities (e.g., people with addictions), and how they missed the opportunities to promote their and their children’s health and well-being.

Consequences on health outcomes

Healthcare providers communicated the evident impacts of the pandemic on the health outcomes of pregnant and parenting youth and their children, be it physical, social, or psychological health. The abrupt disruption of health services and the inability to continue routine appointments during pregnancy and postpartum led to unfavorable consequences on the health of individuals:

A lot of them [youth] were not doing well and were not looking healthy. They stopped attending services. Some had a lot of issues because they weren’t coming into the health center to get care, checkups, routine drugs, especially for new moms that knew nothing about pregnancy. No medication, no right nutrition. So a lot of them were looking so much malnourished. Some women had miscarriages. A lot of them had deformities when they gave birth.

(P-02, nurse)

These health impacts were also precipitated by the stress and anxiety from the pandemic, along with the financial and economic struggles that forced families to limit their expenditure: “I found that they are mostly malnourished because they have not been sleeping well, they have been worried. The anxiety, depression, the stress” (P-13, nutritionist).

The majority of the healthcare providers observed increased reports of mental health issues among their clients, including anxiety, depression, and substance use resulting from fear and uncertainty regarding the future, increased parenting responsibilities, and loss of support networks. The isolation resulting from the lockdowns aggravated mental health concerns, resulting in emotional breakdowns: “In my practice specifically, I also noticed in terms of postpartum and parenting there was higher rates of loneliness and postpartum depression and anxiety” (P-12, nurse).

Participants also talked about the social and developmental impacts on children, with concerns about prolonged isolation, delayed language development, and lack of socialization. Participants indicated that children’s social skills and developmental milestones may potentially be delayed: “Delays or maybe not reaching developmental milestones, because those resources look different, the services look different.” (P-22, social worker)

Deepening vulnerabilities among youth

Along with disrupting the regular sources of support and services, the pandemic exacerbated the existing vulnerabilities among youth, who were susceptible to negative outcomes. As a result of the challenges associated with early pregnancy, financial instability, and societal stigma, some youth were further pushed away from essential services:

The acuity and the number of issues that these parenting teens are facing are just so much worse… the issues are more significant, because they’re less connected, and things have sort of festered for a really long time.

(P-01, nurse)

Several participants commented on the disconnect between vulnerable youth and community, health, and social services during the pandemic, which heightened the insecurities within this population. A provider who worked with vulnerable pregnant youth (e.g., those experiencing homelessness and people with addictions) in the community recalled how the marginalized youth were further segregated from services as a result of the pandemic:

The amount of encampments that started to pop up, and then people living in those encampments weren’t able to go to get support anymore, and people weren’t coming to them. And so it was quite isolating, and a little bit scary, especially when you’re pregnant, right? So I was seeing people, you know, much later in their pregnancies, like sometimes close to delivery, having had no prenatal care, and really, you know, really vulnerable.

(P-11, nurse)

Many providers recognized that it took extra effort to develop connections with youths that would motivate them to seek support and services. These efforts usually worked face-to-face where they could communicate with them, explore their issues, and connect them with the community and resources. However, with social restrictions and remote consultations, these connections became difficult to maintain. Moreover, some participants observed increased intimate partner violence among vulnerable youth during the pandemic, which could have been exacerbated by the inability to seek help and the disruption of healthy family dynamics:

I know that there was a rise in anxiety overall, whether it be because of the pandemic and fear of what the virus was, and understanding how that would impact their pregnancy, or overall stressors, feeling alone, and having to parent alone, if they didn’t have a partner or a supportive family involved… In the folks I’m servicing, since the last few years is more and more challenges and complexities around intimate partner violence, or not having that connection with other people to talk to about power and control within the household, or with their partner.

(P-10, social worker)

Availability and accessibility to quality care

Healthcare providers commented on the youths’ issues while accessing health services within an overwhelmed and resource-exhausted healthcare system. These issues were significant among pregnant youth, who were expected to get regular antenatal care but were unable to secure appointments with the provider of their choice, and faced delays, cancellations, and long waiting times: “There was a reduction in their appointments, keeping up their appointments…it was a barrier because they need these services, and an appointment wasn’t available at the time” (P-24, nurse).

Participants also indicated that fear and anxiety around the virus prevented youth from accessing services; with some afraid of its consequences on their child, and others concerned if they would be forced to vaccinate. Many providers also expressed their discontentment with the youth’s non-compliance with scheduled appointments/visits. Some youth did not have reliable means of transportation to access the health services, which made it even more challenging for them to reach the facility:

Most of them, were complaining of transportation. The means of them getting to the clinics was difficult, because a lot of them wouldn’t want to take a public transport so they don’t get infected.

(P-02, nurse)

Another participant echoed similar concerns:

Teens were accessing healthcare services less. When people came to see us, it was later than when they should have, right? So from a patient perspective, they had maybe infections that were more significant than they would have been if they’d addressed it earlier, or unwanted pregnancy scares, where maybe they didn’t handle it as well as they would have.

(P-01, nurse)

Although many services switched to remote consultations which helped youth to maintain the continuity of care and provided opportunities to connect with health professionals; these services were not ideal for individuals with fewer resources, lower socio-economic status, or crowded households:

Some folks didn’t have reliable access to the Internet, or didn’t have a tablet or a laptop to access. Staying on device was challenging for over an hour, especially with young infants.

(P-10, social worker)

#### 3.2.3. Leveraging Challenges into Opportunities for Service Provision

We examined healthcare providers’ views on service provision challenges and how those challenges were addressed to improve access to services for youth. Participants highlighted the implementation of technological adaptations and their role in exploring different ways to assist youth, investigate their problems, and keep them connected to services.

Benefits and shortcomings of remote consultations

Many healthcare providers, especially those offering antenatal/postnatal programs, mental health services, or social support services, indicated that they switched their operations to virtual consultations completely. Participants said that although the switch was unanticipated and unappreciated at first, it introduced flexibility in consultations, giving pregnant and parenting youth the chance to access services at their own convenience: “The flexibility that has now come with virtual meetings, and it being more seen as an acceptable method going forward” (P-15, psychologist)

Although remote consultations offered ease in accessing services, some healthcare providers communicated that these programs did not work quite as well with youth. They reported that youths did not feel comfortable communicating virtually and were concerned about the privacy and confidentiality of the information shared in group sessions:

A lot of patients [youth] were not able to express what they were feeling, or talk to you freely, because probably there are some things that want to share with you, in your office in person that they wouldn’t like to share it in group conversation. So, it was also still difficult even with that, to get a lot of them to express their feeling or to tell you what they’re feeling. It was also difficult because for some questions, they want to have that privacy

(P-17, psychologist)

Healthcare providers also shared that virtual consultations gave them a chance to obtain insights of what was going on in their client’s lives. They were able to observe the reality of their living conditions and how they maintained their health and well-being. One provider indicated how one participant’s hesitation to attend a virtual appointment revealed the issue of domestic violence:

So it was like their living conditions, it also allowed us to see if there was, issues of domestic violence at home. I remember offering someone a virtual appointment, and they said they were not able to do virtual appointments, and then as we delved further into it, it turns out that there was issues of domestic violence.

(P-12, nurse)

Adaptations of services to improve outcomes

Healthcare providers shared strategies they adopted to continue engaging with young people and provide them with services. This included virtual appointments, community or porch visits, extra clinics, and home check-ins to evaluate their client needs and respond accordingly. Many providers dropped off supplies and resources at their clients’ homes during the pandemic: “The most effective things we did were staying open, being flexible, and trying to deliver service in new ways”. (P-01, nurse).

One provider working in an antenatal clinic, which remained operational, indicated the measures they adopted to accommodate their clients and enhance their comfort:

Trying to just adapt to a person’s situation, meeting them wherever they are, just being very flexible, looking for different options, if they have some fears around the healthcare system, what can we do to accommodate that? Like a nurse coming out, or maybe even connecting them to like a midwife instead

(P-07, nurse)

Moreover, participants identified a notable shift towards equity and inclusivity in healthcare discussions, with increased recognition of the importance of serving marginalized communities. This shift reflected a broader societal conversation surrounding the need for equitable access to healthcare services: “There’s been a lot more conversations across healthcare landscapes… there’s a shift to be able to recognize the importance of ensuring that we don’t leave certain communities and folks behind” (P-10, social worker).

Some providers also came up with innovative strategies, such as creating YouTube videos, for their clients to continue to give them awareness and education about their needs:

I would record weekly YouTube videos. It’s like one week we’d do a prenatal topic, the second week we’d do a relapse prevention, addiction and recovery related topic. It would be me presenting. I’d show a video or something. I’d talk about withdrawal, I’d talk about what to look for, and then just kind of end it with, “And if you need to call me, please call me, and we can discuss it further”

(P-11, nurse)

Recommendations for future crises

We asked participants to recommend strategies and interventions to continue supporting pregnant and parenting youth during crises like the pandemic. Healthcare providers recommended several strategies, such as improved governmental funding for services, educational programs for youth, and comprehensive antenatal and postnatal care initiatives for vulnerable youth. Participants highlighted that youths may need assistance and guidance with the appropriate and effective use of virtual platforms to seek help and access resources in cases of emergencies: “I would suggest proper education on how to access and utilize video conferencing meetings” (P-03, social worker).

Participants also stressed the importance of connecting vulnerable youth to a community of resources to improve their engagement with other individuals:

I think a big part afterwards, at least, is finding ways to reconnect them to the community, because that part is missing a lot. So whether that be reconnecting to community resources, whether that be helping, and getting them access to places where they can now be a part of the community.

(P-15, psychologist)

Participants recommended a more integrated approach to healthcare services for pregnant and parenting youth. One healthcare provider suggested providing youth with an opportunity to address their and their children’s needs all in one place:

I would also advocate to ensure clinics that are multidisciplinary, to help this patient population. So for example, having a perinatal clinic that has a social worker, dietitian, a physician, maybe a psychiatrist as well, who specializes in this area. And maybe even a pediatrician. Like having like a one-stop shop for these folks, under one umbrella, or like one area where they can do multiple appointments.

(P-12, nurse)

## 4. Discussion

We present an exploration of the perceptions of healthcare and service providers on how the COVID-19 pandemic influenced care provider roles, delivery of health and social support services, and programs dedicated to pregnant and parenting youth in Canada. We found that the healthcare providers in our study faced significant challenges while providing care to pregnant and parenting youth, with many experiencing burnout and stress associated with overwhelming public health policies, restructuring of the healthcare system, and an increase in workload.

Pregnant and parenting youth are a vulnerable population, whose access to perinatal services, social support services, and the healthcare system was severely affected during the active stages of the pandemic [22,23,24]. The healthcare professionals in our study reported the sudden halt of access to routine services (e.g., antenatal/postnatal appointments) which, combined with the loss of support networks, negatively impacted the overall well-being of pregnant and parenting youth and their children. They also indicated higher incidences of anxiety, depression, and mental health disorders among their patients, which was reported in several studies globally [23,24,25,26]. Perinatal care providers in the U.K. shared similar concerns regarding young parents and how they were found to be more prone to negative outcomes, given their socio-economic vulnerabilities, lack of access to information and support, and increased risk for complications [23]. Moreover, providers in our study witnessed the exacerbation of vulnerabilities among marginalized youth (e.g., substance use, and homelessness) during the pandemic. This further distanced them from essential services and made them dubious of ever receiving perinatal care throughout their pregnancy. Providers also expressed their apprehensions about youths experiencing issues of intimate partner violence while being confined to their residences during lockdowns. A scoping review by Kotlar et al. [22] reported notable increases in the cases of domestic violence in many countries during the pandemic, which can have deleterious effects on maternal health outcomes.

Participants reflected on the various policies, adaptations, and system changes that were implemented to prevent the spread of the virus, and its consequences on their working conditions and patient outcomes. First, the restriction on the presence of a family member, partner, or support person during childbirth was challenging for younger mothers, as they were unable to receive support and guidance during a critical time and share this moment with their partner. Many studies have reported similar emotional distress among pregnant women who had to give birth alone and have documented consequences on their emotional and mental health, especially considering the instrumental role of support companions in determining pregnancy outcomes [22,23,24,25,26,27,28]. In such situations, perinatal support workers and health professionals were seen to assume social and emotional support roles to provide comfort to distressed clients [23,24,26]. Second, limitations on the number of patients visiting healthcare facilities impeded the ability of pregnant and parenting youth to effectively interact with their healthcare providers, follow up on their regular appointments, and seek information or guidance for concerns (e.g., breastfeeding). To lower the patient ratio and reduce chances of exposure, length-of-stay in the hospital postpartum was significantly reduced and, in some cases, caesarean sections were performed as a preferable mode of childbirth during the pandemic [22,26,29,30]. Such measures led to social and emotional isolation, feelings of neglect by the healthcare system, and unpreparedness to care for a newborn, which also aligns with other literature in the field [22,23,26]. Lastly, the reduction in face-to-face time and limited opportunities to connect in various capacities (e.g., home visits, and antenatal checkups) significantly altered the care dynamics, with many healthcare providers citing difficulties in building rapport and trusting relationships with clients, which was of most importance with youth. Healthcare providers across Europe, Canada, and the U.K. have cited similar challenges due to the influence of physical distancing, virtual care, and reduced in-person appointments on the quality of patient–provider relationships, with many providers feeling distanced from their patients [22,23,24,29].

The enhanced measures for the pandemic response (e.g., PPE, distancing, redeployment) combined with the impediments to providing compassionate and quality care, caused stress, burnout, and emotional exhaustion among healthcare providers. The abrupt alteration of the healthcare environment, rapidly changing policies, and ensuring continuity of care while simultaneously minimizing exposure for themselves and patients led to significant frustration among care providers [24,27,29]. A global survey conducted with 714 perinatal healthcare professionals reported that most providers (81%) noted their work being impacted by the pandemic, whereas the stress levels for 90% of the respondents were higher than usual [30]. The literature suggests that health institutions and governments were unprepared for the pandemic’s rapid expansion and that maternal and newborn healthcare may not have been a top priority [31]. However, recognizing the importance of continuity of care, perinatal services quickly adapted the use of technology to continue to offer services. Many providers in our study leveraged technology in several ways, such as to develop informational content, or organize group sessions (e.g., birthing classes). While providers acknowledged that the technology helped them to continue their patients’ plan of care and address their concerns as best they could, it was still not an alternative to face-to-face interactions. This was particularly true for youth, as providers faced difficulty engaging youth through virtual platforms, citing hesitancy, technological problems, and lack of access to devices (e.g., laptops, and smartphones). Studies have found varying levels of success and effectiveness of telehealth interventions, with some reporting the ease of consultations, reducing unnecessary visits, and conveniently providing information over the phone [24,31]; whereas others have reported potential drawbacks, such as the inability to promptly diagnose a condition that required comprehensive assessments and inability to reach marginalized individuals [23,24,29]. The mass implementation of telemedicine for maternal and newborn healthcare may have potentially increased the existing inequalities and disparities in access to high-quality care among vulnerable groups [31], which points to the necessity of research to develop evidence-based guidelines for effectively implementing such measures in crises.

### 4.1. Implications and Future Research

Our findings have important implications for healthcare providers, policymakers, and practitioners in the field. Healthcare providers in our study acknowledged the necessity to implement strict precautionary measures to control the disease; however, they also stressed the consequences of such policies, emphasizing the need for alternate plans of care for maternal and child health for young parents during crises. As maternal health can quickly deteriorate during the term of pregnancy, virtual appointments may not be sufficient enough to rule out any abnormalities or advanced care requirements. Evidence-based guidelines and policies are required to ensure the continuity of care for this vulnerable group during such emergencies. Healthcare providers recommended the integration of health services to cater to the different needs of youth and their children in one holistic setting. Since the peak of the pandemic, there have been adaptations in service delivery models, such as the increased integration of virtual care into routine healthcare services. However, challenges persist, particularly for marginalized groups, like pregnant and parenting youth, who often face barriers to accessing technology and maintaining solid connections with healthcare providers in a virtual setting. Our findings remain relevant as they highlight these ongoing issues and inform the development of more equitable and sustainable healthcare models. The persistent challenges strengthen the need for the recommended changes, such as improving access to technology, providing flexible care options, and ensuring continued support during public health crises. Moreover, participants in our study also mentioned significant cutbacks in funding for maternal health programs and services (e.g., community health programs, and social programs), which made it difficult to continue providing services and care to vulnerable youth. Governments and policymakers need to develop contingency plans that ensure continued support and funding for such programs, especially during public health emergencies. Specific measures could include establishing emergency funding reserves for maternal and child health services, particularly for vulnerable populations, such as pregnant and parenting youth. Additionally, governments could implement flexible service delivery models incorporating in-person and virtual care options to maintain service continuity during crises. Developing robust telehealth infrastructure and providing technological resources (e.g., internet access and devices) to marginalized communities would also help ensure equitable access to care. Lastly, the rapidly changing policies made it difficult for healthcare providers to adequately respond to the needs of pregnant and parenting youth while following the policies and keeping up-to-date with accurate information. Therefore, there is a need to develop interventions and knowledge translation tools (e.g., toolkit) for healthcare providers which can serve as a guide to advocate for pregnant and parenting youth and their children in future events.

### 4.2. Strengths and Limitations

While studies have targeted healthcare providers working with pregnant women, to our knowledge, this is the first study in Canada to investigate healthcare providers’ experiences working with pregnant and parenting youth during the pandemic. This unique perspective gives us important insights to understand the necessary services and support that youth usually require but are unable to access due to several challenges and structural barriers. We included healthcare providers from diverse fields and specialties across three major provinces in Canada, which helped us to obtain a holistic understanding of the issues that youth encountered.

Despite including providers from diverse fields, one of the limitations of our study is regarding the generalizability of our findings. Although the sample size was sufficient to achieve meaningful saturation, the sample may not represent Canada’s entire healthcare and community services workforce. This may limit the applicability and generalizability of the findings to specific contexts. Healthcare providers in rural settings may have had different experiences and challenges during the pandemic, which were not significantly highlighted in our findings. Similarly, the findings may not broadly apply to different healthcare systems worldwide and can be interpreted as context specific. Further research can build upon these findings to include more geographically diverse populations to compare how the experiences of healthcare and workers differed across different settings and systems. Lastly, we conducted all the interviews over Zoom, which limited our understanding of how participants felt when they talked about their experiences. This could have led to not articulating their issues accurately.

## 5. Conclusions

The emergence of the pandemic and its associated policies significantly impacted the delivery of health services and access to quality care, particularly for vulnerable populations, such as pregnant and parenting youth. While healthcare professionals and service providers identified several barriers and challenges, our findings also offer broader implications for improving healthcare models beyond the pandemic. Some of the challenges were associated with the inability to receive adequate support and quality care from the healthcare system, which ultimately led to youth being distanced. Healthcare providers also reflected on how the policies and rapidly changing guidelines increased their stress levels, added more workload, and limited opportunities to adequately attend to their clients’ needs. The opportunities that arose from the implementation of telemedicine helped to ensure that youth have access to essential services and resources. However, participants also commented on the equitable use of such interventions and designing policies and strategies that target vulnerable groups and help them gain access to care during emergencies. These insights can inform policies and practices to enhance resilience and adaptability in healthcare systems, ensuring continuity of care for vulnerable groups during future public health emergencies. Practical applications of our findings include integrating flexible service delivery models, addressing barriers to virtual care, and improving equitable access to essential healthcare services. These revisions can help healthcare providers, policymakers, and researchers design more responsive and inclusive care models that resonate with a broader audience and have long-term impacts on maternal and child health.

## Figures and Tables

**Table 1:** Demographic Characteristics of Participants (*n* = 25).

Characteristics	*n*	*%*
**Service Provider Role**		
Nurse	10	(40)
Midwife	1	(4)
Pharmacist	2	(8)
Physician	2	(8)
Social worker	5	(20)
Psychologist	3	(12)
Nutritionist	2	(8)
**Location (Province)**		
Alberta	15	(60)
Ontario	8	(32)
British Columbia	2	(8)
**Years of Experience**		
1–5 years	9	(36)
6–10 years	9	(36)
11–15 years	2	(8)
16–20 years	5	(20)

## Data Availability

The original contributions presented in this study are included in the article/Supplementary Material. Further inquiries can be directed to the corresponding author.

## Supplementary Materials

The following supporting information can be downloaded at: https://www.mdpi.com/article/10.3390/ijerph21111419/s1.
