# Supplementary material for: Healthcare Provider Narratives of the Impacts of the COVID-19 Pandemic on Pregnant and Parenting Youth in Canada: A Qualitative Study
Source: Int J Environ Res Public Health. 2024 Oct 26;21(11):1419. doi: 10.3390/ijerph21111419 (PMC11594201; doi:10.3390/ijerph21111419)
Supplement: Supplementary file 1 [file ijerph-21-01419-s001.zip › ijerph-3243011-supplementary.pdf]

<u>Interview Guide for Service Providers (draft)</u>

Impact of the COVID-19 Pandemic on Sexual and Reproductive Health of Adolescents in Alberta

<u>Initial Open-Ended Questions</u>

1. How long have you been working as a SRH service provider?
2. What has been your experience as a provider working with adolescents before and during the pandemic? (have you notice any change in number of patients/adolescents come to access the services during pandemic?)
3. How has the pandemic influenced adolescents' access to SRH services?
4. What are the challenges you have faced in providing SRH services during the COVID-19 pandemic? (Probing questions: Access to in-person services and walk-in services, online services, redeployment of staff.)
5. Did you experience any challenges using digital technology to provide services remotely? (like privacy issue, non-compliance with appointments by adolescents)
6. How did staff shortage and social distancing have an effect on regular access to SRH services by adolescents?
7. Were you able to take in new patients during the pandemic?
8. What was the most effective strategy you were able to develop during the pandemic to provide SRH services to adolescents? Or to improve the access to SRH services to adolescents?
9. What you think how we can best support adolescents to access SRH services during and after pandemic or in any future pandemics?
10. Anything else you want to add which I missed to ask you?
